# Modulation of endogenous antioxidant defense and the progression of kidney disease in multi-heritage groups of patients with type 2 diabetes: PRospective EValuation of Early Nephropathy and its Treatment (PREVENT)

**DOI:** 10.1186/s12967-016-0975-9

**Published:** 2016-08-04

**Authors:** Kenneth A. Earle, Karima Zitouni, John Pepe, Maria Karaflou, James Godbold

**Affiliations:** 1 St Georges University Hospitals NHS Foundation Trust, Blackshaw Road, London, SW 17 0RE UK; 2 Clinical Sciences Division, St. Georges University of London, London, UK; 3 Department of Preventive Medicine, Icahn School of Medicine at Mount Sinai, New York, USA; 4 Richmond University, Staten Island, New York, USA

## Abstract

**Background:**

Diabetes is the western world’s leading cause of end-stage renal disease. Glucose-dependent, oxidative stress is linked to the development of renal inflammation and sclerosis, which, in animal models of diabetes, can be prevented by anti-oxidative treatment. Patients of non-Caucasian heritage have low activity of the selenoprotein, antioxidant enzyme, glutathione peroxidase (GPx) and its co-factor vitamin E, which may be linked to their increased propensity to developing end-stage renal disease.

**Research design and methods:**

We have designed a double-blind, randomized, placebo controlled study with selenium and/or vitamin E versus placebo as the interventions for patients with type 2 diabetes and chronic kidney disease (CKD) stages 1–3. A 2 × 2 factorial design will allow a balanced representation of the heritage groups exposed to each intervention. The primary biochemical outcome is change in GPx activity, and clinical outcome measure is the actual, rate of—and/or percentage change in estimated glomerular filtration rate (eGFR) from baseline. Analysis will be with a marginal model for longitudinal data using Generalized Estimating Equations corrected for measures of baseline serum antioxidant enzyme activities (GPx, superoxide dismutase and catalase), micronutrient levels (vitamins E and C), measures of inflammation (interleukin 6, c-reactive protein and monocyte chemoattractant protein-1) and markers of oxidative damage (plasma 8-isoprostaglandin F2α and urinary 8-hydroxydeoxyguanosine).

**Expected results:**

The study will assess the relationship between GPx activity, oxidative stress, inflammation and eGFR. It will test the null hypothesis that antioxidant therapy does not influence the activity of GPx or other antioxidant enzymes and/or alter the rate of change in eGFR in these patient groups.

**Conclusions:**

Outcome data on the effect of antioxidants in human diabetic renal disease is limited. Previous post hoc analyses have not shown a beneficial effect of vitamin E on renal function. A recent trial of a pharmaceutical antioxidant agent, improved eGFR, but in patients with advanced diabetes-related chronic kidney disease its use was associated with an increased incidence of cardiovascular events. We will explore whether the nutritional antioxidants, vitamin E and selenium alone, or in combination in patients at high risk of renal disease progression, forestalls a reduction in eGFR. The study will describe whether endogenous antioxidant enzyme defenses can be safely modified by this intervention and how this is associated with changes in markers of oxidative stress.

*Trial registration* ISRCTN 97358113. Registered 21st September 2009

## Background

Diabetes is the leading cause of end-stage renal disease (ESRD) which has a predilection for persons of non-white heritage [1]. Comparative international, and national studies suggest that the rate of progression of chronic kidney disease (CKD) is higher in patients of non-white heritage than those of Caucasian heritage [1–3]. In a post hoc analysis of the United Kingdom Prospective Study, an independent relationship was found between non-white ethnic heritage, albuminuria and renal impairment [4]. These observational data suggest that heritable and/or shared environmental factors play a role in the susceptibility to, and progression of renal disease in patients with diabetes.

Hyperglycaemia increases the production of reactive oxygen species (ROS) which are capable of damaging lipid and DNA molecules. Oxidative stress occurs when ROS production overwhelms the host’s capacity to remove them which then causes upregulation in transcription factors such as NF-κB and elaboration of pro-inflammatory cytokines and chemokines. These biochemical events limit the availability of the vasodilator nitric oxide, which interferes with the regulation of blood flow and vasoprotective functions of the endothelium [5]. In experimental studies, these changes have been shown to be determinants of the development of diabetic kidney lesions which can be ameliorated with antioxidant treatment. In a rodent model of type 2 diabetes and kidney disease, vitamin E given as an antioxidant therapy, restored antioxidant enzyme activity and reduced oxidative damage of renal tissue [6]. This has led to suggestions that strategies to enhance endogenous antioxidant mechanisms could help prevent microvascular diabetic complications [7].

Markers of oxidative stress are increased in the circulation and renal tissue of patients with diabetes compared with control patients without diabetes [8, 9]. Patients of African origin have been found to have higher circulating levels of lipid peroxide, lower concentrations of vitamin E and faster rates of decline in renal function compared with those of Caucasian heritage [10, 11]. Relatively low levels of serum selenium have been reported in people of Asian origin [12]. Selenium is a co-factor for the antioxidant enzyme GPx, and low levels are associated with an upregulation of inflammatory chemokines. Selenium supplementation attenuates the inflammatory response and in systemic inflammatory disease states may improve clinical outcomes [13, 14].

The activity of GPx in plasma is largely derived from renal tubular epithelium and is reduced in the presence of renal disease [15, 16]. It is unclear whether the level of activity of GPx contributes to the susceptibility to progressive renal disease. In this study, we will compare the effect over time of antioxidant therapy with vitamin E and/or selenium in patients with type 2 diabetes and early CKD, on measures of systemic inflammation, antioxidant enzyme activity and changes in eGFR.

## Subjects

**Fig. 1:**
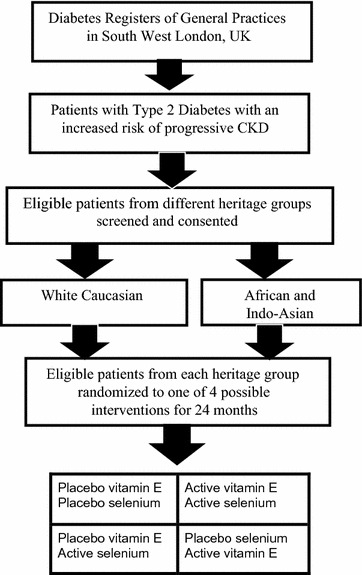
Flow diagram of the recruitment, randomization and follow-up of eligible patients double-blind, randomized placebo controlled trial into the 2 × 2 factorial, to achieve balanced representation of non-Caucasian and Caucasian heritage groups

We plan to recruit a total of 200 adult patients with type 2 diabetes of white, Caucasian and non-Caucasian (African, Caribbean or Indo-Asian) heritage from general practices in South West London, UK. An equal number of eligible patients will be randomized to receive, either, active selenium (200 μg once daily) or its placebo and/or vitamin E (400 IU once daily) or its placebo in a 2 × 2 factorial design (Fig. 1). Treatment allocation will be according to a computer generated code for each group to which the patients and researchers will be blinded.

Type 2 diabetes mellitus will be diagnosed according to WHO criteria. Eligible patients will have hypertension (3 consecutive sitting blood pressure readings >140 systolic and/or diastolic 90 mmHg without treatment or receiving treatment for known hypertension) and early CKD defined as an eGFR > 45 and <90 mL/min/1.73 m^2^ and/or urinary albumin:creatinine ratio >3 mg/mmol.

Patients will be excluded if they have any of the following: a history of cardiovascular disease, defined as having a clinical record of ischaemic heart disease (angina, myocardial infarction, coronary artery revascularization and or heart failure), peripheral vascular disease (intermittent claudication or peripheral artery revascularization) or cerebrovascular disease (transient ischaemic episodes or stroke), a history of malignancy or any other life threatening illness, current pregnancy, systolic blood pressure >200 mmHg, diastolic blood pressure >160 mmHg, haemoglobin A1c > 86 mmol/mol (10 %), significant renal impairment (eGFR < 45 mL/min 1.73 m^2^) and nephrotic range urine protein excretion (total protein excretion rate >3 g/day or albumin:creatinine ratio >300 mg/mmol).

The medical, family, social and treatment histories, and the clinical and biochemical measurements collected at the in-person visits will be recorded using a standardized, electronic proforma. Dietary intake and patterns will be evaluated using a 3-day food diary to assess components of the usual diet (Dietplan 6, Forestfield Software Ltd, Horsham, West Sussex, UK).

## Methods

Anthropometric measures will include height in metres, weight in kilogrammes, and waist circumference in centimetres. Body mass index will be calculated from the weight in kilogrammes divided by the height in metres squared. Sitting blood pressure will be measured by digital oscillometry (Omron 705IT, OMRON Healthcare Europe, The Netherlands) according to the National Institutes of Health and Care Excellence guidance (http://www.nice.org.uk/guidance/cg127).

Patients will undergo routine clinical assessments for the complications of diabetes. The presence or absence of retinopathy will be reviewed on an annual basis by standardized, digital retinal fundal photography which will be reported according to National Health Service guidelines by retinal screeners who are unaware of the patient’s participation in the study (http://www.diabeticeye.screening.nhs.uk/gradingcriteria). Peripheral neuropathy will be assessed according to responses to testing with a 10 g monofilament. Peripheral vascular disease will be assessed by the appreciation of the dorsalis pedis and posterior tibial artery pulsation by digital examination. Patients will also be screened for ischaemic heart disease in response to modified questions from the Rose questionnaire [17] and have 12-lead resting electrocardiography (Seca CardioConcept 5.6, Seca UK) performed and read using Novacode criteria [18].

### Arterial stiffness

Finger plethysmography is a non-invasive method to assess changes in finger blood flow that will be used as a proxy measure of arterial stiffness. The pulse wave amplitude will be measured from the finger-tip using infra-red light (PulseTrace PCA2, CareFusion UK 232 Ltd, Basingstoke). The contour of the finger pulse will then be automatically analysed to determine a stiffness index which has been shown to be correlate with pulse wave velocity after adjustment age, sex, height, and weight [19].

### Biochemical assessments

Venous blood will be sampled after a 12 h fast. Measurements will be performed to allow several domains of the study to be addressed, including lipids, systemic inflammation, oxidative damage and stress, diabetes control and nutrition. Deoxyribonucleic acid (DNA) will be extracted from whole blood using a salting-out method as described by Miller et al. [20] for the subsequent analysis of genetic polymorphisms of the antioxidant enzymes being investigated.

Plasma creatinine will be used to estimate renal function—using the modification of diet in renal disease (MDRD) and CKD-EPI equations. Three early morning urine samples will be collected for estimation of the median albumin: creatinine ratio. Aliquots of the urine, whole blood and blood separated into serum and plasma will be stored at −70 to −80 °C for later analysis of the key outcome variables described below.

Glutathione peroxidase activity will be measured as described previously by Zitouni et al. [11]. In brief, 500 μL Tris–HCl (0.2 mmol/L, pH 8) will be mixed with NADPH (0.2 mmol) followed by 100 μL EDTA (5 mmol/L) and 100 μL glutathione (20 mmol/L). Plasma (100 μL) will then be mixed with glutathione reductase (0.002 units) and incubated for 5 min at 37 °C and for a further 5 min after the addition of 100 μL of tert-butyl hydroperoxide (0.7 mmol/L). Absorbances will be read in a spectrophotometer at 340 nm. A unit of GPx activity is defined as being equivalent to the oxidation of 1 μmol of NADPH per second at 37 °C. The intra-assay coefficient of variation (c.v.) is 3.2 %.

Catalase activity in plasma will be measured using a fluorometric assay. Plasma (50 μL) will be mixed with 400 μL phosphate buffer (100 mmol, pH 7) followed by 50 μL horseradish peroxidase (40 U/ml) and 100 μL of hydrogen peroxide (3.3 μmol/L). The samples will then be vortex-mixed and incubated for 10 min at room temperature. After incubation, 50 μL of hydroxyphenyl acetate (50 mg/mL) will be added and the fluorescence of the product will be read in a fluorometer (excitation 320 nm and emission 400 nm). Within-run c.v.’s are 2 % for standards and samples, whereas the between-run c.v. are 3.1 % for standards and 7.3 % for samples.

Extracellular SOD activity will be measured in relation to the inhibition of the rate of nitro blue tetrazolium (NBT) reduction in the presence of xanthine and xanthine oxidase yielding a purple colour [11]. Plasma (500 μL) will be treated with 300 μL chloroform and 500 μL ethanol. The samples will then be centrifuged at 18,000 × *g* for 30 min, and 50 μL of the supernatant will be removed and mixed with 900 μL of SOD reagent [0.1 mmol/L xanthine, 0.1 mmol/L EDTA, 50 mg bovine serum albumin, 25 mmol/L NBT and 40 mmol/L Na_2_CO_3_ (pH 10.2)]. Twenty-five units of xanthine oxidase will then be added, and the samples will be incubated for 20 min at 25 °C. The reaction will be stopped by the addition of 1 mL CuCl_2_ (0.8 mmol/L), and the absorbance of the samples will be measured at 560 nm. The intra-assay c.v. is 3.5 %.

Vitamin E as plasma α- and γ-tocopherols will be measured using a high-performance liquid chromatography system with ultraviolet detection and tocopherol acetate as internal standard. Briefly, plasma (100 μL) will be combined with 3 μg α-tocopherol acetate and 500 μL hexane. The upper layer will be aspirated after centrifugation, and lipid extraction will be performed twice. The solvent will be evaporated to dryness under a stream of nitrogen. The residue will be dissolved into 400 μL methanol and 20 μL will be injected onto the high performance liquid chromatography system. α-Tocopherol and γ-tocopherol concentrations in the samples will be calculated by relating their peak areas to that of the internal standard. The plasma contents of absolute and lipid standardized vitamin E will be expressed relative to triglyceride, cholesterol, or triglyceride and cholesterol.

The total cholesterol content of plasma will be measured by an enzymatic colorimetric method (Cholesterol CHOD-PAP; Sigma Diagnostic). Briefly, an aliquot of plasma sample (10 µL) will be incubated with a reagent (900 µL) containing: detergent, cholesterol esterase, and cholesterol oxidase. Hydrogen peroxide produced as a result of cholesterol oxidase activity is used to oxidise aminoantipyrene to give a pink product, the absorption of which will be measured in a spectrophotometer at 500 nm.

The total triglyceride contents of plasma and lipoprotein fractions will be measured by an enzymatic colorimetric method (Triglycerides GPO-PAP; Sigma Diagnostics). In brief, an aliquot of plasma sample (10 µL) will be mixed with 900 µL of a reagent containing a lipase to deacylate triglycerides and liberate glycerol. This will then be phosphorylated to glucose-1-phosphate and the product oxidised by glucose phosphate oxidase with the formation of hydrogen peroxide (H_2_O_2_). The H_2_O_2_ formed will then be coupled with horseradish peroxidase to give a pink product, the absorption of which will be measured in a spectrophotometer at 500 nm.

Vitamin C as total ascorbate (sum of oxidised and reduced ascorbate) will be measured by enzymatic oxidation of reduced ascorbic acid to dehydroascorbate acid followed by reaction of the dehydroascorbate formed with o-phenylenediamine to give a fluorescent product [21]. Two hundred and fifty microliters of plasma will be mixed with 250 μL metaphosphoric acid (20 % w/v) and centrifuged at 13 000×*g* for 5 min. 50 μL of the supernatants will be combined with 900 μL of sodium acetate buffer (2 mol/L, pH 6.2) and ascorbate oxidase (10 μL of 1 U/1 μL in sodium acetate buffer). o-1, 2-diphenylenediamine (20 μL, 1 mg/mL) is then added and the samples are incubated for 30 min at room temperature in the dark. Finally, the fluorescent signals will be read in spectrofluorometer at excitation at emission wavelengths of 350 and 430 nm, respectively. The inter- and intra-assay c.v. are 1 and 2.5 %, respectively.

Selenium will be measured in serum as described by Rayman et al. [22]. The analysis will be carried out at the SAS Trace Element Unit, Southampton General Hospital using dynamic reaction cell-inductively coupled plasma MS on an Elan 6100 DRC plus (SCIEX Perkin-Elmer).

Oxidative stress will be assessed by measuring (1) plasma and urinary free 8-iso prostaglandin F2α using an ELISA kit (Cayman Chemical, Ann Arbor, MI, USA) and (2) urinary 8-hydroxydeoxyguanosine (8-OHdG) as a marker of deoxyribonucleic acid damage using an ELISA kit according to the manufacturer’s instructions (Cambridge Biosciences Ltd, Cambridge, UK).

Inflammation will be assessed by measuring c-reactive protein, interleukin-6 and monocyte chemoattractant protein-1 by ELISA kits according to the manufacturer’s instructions (ThermoFisher Scientific, Paisley, UK).

### Clinical follow-up procedures

All patients will receive their usual care according to the latest National Institutes of Clinical Excellence recommendations, in primary and secondary care settings [23]. Treatment will aim to achieve targets for blood pressure of ≤130/80 mmHg, glycated haemoglobin of 59 mmol/mol (<7.5 %) and LDL-cholesterol of ≤2 mmol/L (<100 mg/dL). Routine clinical and biochemical measurements and outcome assessments will be performed at the baseline visit and 1, 3, 6, 12, 18 and 24 months after randomization. All of the patient’s data will be anonymized.

### Statistical analysis

In this 2 × 2 factorial design, selenium and vitamin E are considered the two factors. The data will be analyzed with a linear mixed model for longitudinal data [24]. In the model, eGFR is the dependent variable and time is an independent variable; the model will also contain as independent variables a dummy variable for each of the two factors, a term for the two-way interaction between the two factors, and continuous covariates for various measures of oxidative stress and inflammation at baseline. The model allows for the correlation between repeated measures on the same individual, but assumes that there is independence from one subject to the next. The correlation within measurements on a given subject will be modeled using compound symmetry as well as an autoregressive correlation structure. Whichever structure gives the best fitting model to the data will be used.

## Results

The primary hypothesis of interest in this study is whether there is an interaction between the selenium and Vitamin E supplements. If interaction is present, then the effect of each supplement will have to be estimated separately in the presence and absence of the other supplement. If no interaction is present, then it will be possible to estimate the main effects of selenium and Vitamin E separately without having to take the other supplement into consideration.

The effect sizes are conservative and will be based on the estimated changes from baseline to 1 year [25]. A study population of 150 will have 85 % power to detect an ES of 1.0 SD, and a population of 200 will have 83 % power to detect an ES of 0.85 SD at a two-sided alpha level of 5 % [26].

The two heritage groups will have their eGFR slopes compared over time and considered as either, (a) Caucasian and non-Caucasian, or (b) High and Low levels of oxidative stress/inflammation, with the oxidative stress and inflammation markers dichotomized at their median value. The power will not be heavily dependent on any of the assumptions; regardless of the true value of the underlying correlation among observations with subjects, of the correlation structure, of the attrition rate, or of the number of repeated measurements.

## Discussion

Chronic kidney disease is a world-wide, major public health concern which, depending upon classification codes affects up to 1:3 patients with type 2 diabetes [27]. Chronic kidney disease is a costly complication of diabetes. In the United Kingdom, it has been estimated that 10 % of its £110 billion ($183 billion USD) healthcare budget is spent managing diabetes and £1.5 ($2.5 billion) on CKD and renal replacement therapy [28].

There has been a reduction in ESRD over the last decade which may be related to better management of traditional risk factors [29]. However, diabetes remains the leading cause of ESRD and has a striking predilection for persons of non-white heritage. The 2014 United Renal Data System reported that in 2012, the incidence of ESRD amongst persons with diabetes of African (non-Caucasian) heritage was fourfold greater compared with those of white (Caucasian) origin. Of interest, the report reveals that between 2007 and 2012 there was a difference of only 2 % in the prevalence of CKD between the groups. Some of the difference in kidney disease progression is explained by the relatively higher rates of hypertension and proteinuria in the patients of African origin, but is unrelated to variations in access to healthcare before dialysis [30]. However, 1:3 patients with diabetes at risk of CKD may not have increased urinary protein excretion [31]. In the United Kingdom Prospective Diabetes Study, in which patients were followed-up from the diagnosis of type 2 diabetes, racial heritage was found to be an independent determinant of renal dysfunction [32]. And, recently, it has been reported that inflammatory stress, has a stronger relationship than albuminuria with early CKD in patients with diabetes of African heritage, compared with other heritage groups [33].

A large experimental evidence base supports the role of inflammatory and oxidative stress in the development of kidney lesions in diabetes. However, clinical data concerning the effect of antioxidant therapy in preventing renal (and vascular disease) is conflicting. Several studies suggest that vitamin E in non-diabetic patients with high levels of oxidative stress has a beneficial impact on hypertension-related disorders and reduces biochemical markers of oxidation [34, 35]. However, post hoc analyses of some antioxidant trials did not show any effect on cardiovascular or renal end-points [36, 37]. It is possible that prevalent, systemic vascular damage might mitigate any benefit of antioxidant treatment. Furthermore, these analyses have not related clinical outcomes to actual measures of vitamin E or other evaluations of oxidant and/or antioxidant status.

There is some evidence that vitamin E suppresses albuminuria in patients with diabetes, preserved renal function and without cardiovascular disease [38]. Moreover, patients with high oxidant stress due to genetically determined low levels of the antioxidant haptoglobin, who received vitamin E, had a significantly reduced incidence of vascular events compared to controls [39]. In a rodent model of type 2 diabetes, treatment with tocotrienol-rich fractions improved glycemic status, serum lipid profile and renal function in association with restoration of anti-oxidant enzyme activity [40]. These data suggest that, oxidative stress promotes renal and vascular damage that may be ameliorated by an antioxidant intervention in those patients at highest risk of their development.

Previously, we found GPx activity to be lower in patients with type 2 diabetes of African heritage compared with Caucasians. Others have shown that low GPx activity is associated with an accelerated development of vascular lesions in both experimental rodent models, and patients with diabetes [41, [42]. However, data are limited on the role of GPx activity or its regulation with respect to the development or progression of renal disease in patients with diabetes. The PREVENT trial will provide new information on whether progression of the early stages of CKD, is related to, or modified by, oxidative stress and/or host antioxidant defense mechanisms in type 2 diabetes.

## Conclusions

In animal models of diabetes, there is overwhelming experimental evidence for a role of oxidative stress in renal pathophysiology. However, most clinical reports of antioxidant therapy have been post hoc analyses of surrogate markers, in non-specific populations with conflicting outcomes and safety concerns. We will primarily address renal functional decline in patients with diabetes at high risk of progressive disease. Importantly, it will investigate whether a nutraceutical approach to modulate an endogenous antioxidant pathway is safe and effective. The outcomes will help improve our understanding of the translation of oxidative metabolism in the development and treatment of renal disease.

## Abbreviations

GPx: glutathione peroxidase
CKD: chronic kidney disease
eGFR: estimated glomerular filtration rate
ESRD: end stage renal disease
ROS: reactive oxygen species
DNA: deoxyribonucleic acid
NADPH: nicotinamide adenine dinucleotide phosphate
